# Volatile Compounds in Monovarietal Wines of Two Amarone Della Valpolicella Terroirs: Chemical and Sensory Impact of Grape Variety and Origin, Yeast Strain and Spontaneous Fermentation

**DOI:** 10.3390/foods10102474

**Published:** 2021-10-15

**Authors:** Giovanni Luzzini, Davide Slaghenaufi, Maurizio Ugliano

**Affiliations:** Department of Biotechnology, University of Verona, Villa Lebrecht, via della Pieve 70, 37029 San Pietro in Cariano, Italy; giovanni.luzzini@univr.it (G.L.); davide.slaghenaufi@univr.it (D.S.)

**Keywords:** grape withering, yeast selection, terroir, spontaneous fermentation, Amarone della Valpolicella, ethyl acetate

## Abstract

Aroma profiles of withered Corvina and Corvinone wines from two different Valpolicella terroirs were investigated in relationship to yeast strain and use of spontaneous fermentation. The results indicated that volatile chemical differences between wines were mainly driven by grape origin, which was associated with distinctive compositional profiles. Wine content in terpenes, norisoprenoids, benzenoids and C_6_ alcohols, as well as some fermentative esters, were indeed significantly affected by grape origin. Conversely, yeast strain influence was mainly associated with fermentation-derived esters. Sensory analysis, besides confirming the major role of grape origin as driver of wine differentiation, indicated that spontaneous fermentations reduced the sensory differences associated with grape origin and variety, mainly due to high content of acetic acid and ethyl acetate.

## 1. Introduction

Valpolicella is an Italian wine region, well-known for the production of premium red wines. A peculiar feature of this region is the widespread use of post-harvest grape withering for the production of red wines, in particular the dry red passito Amarone della Valpolicella. Another characteristic of Valpolicella is the unique blend of grape varieties used for the production of its Protected Designation of Origin (PDO) wines, including the two main varieties Corvina and Corvinone, to be blended along with a range of other minor varieties [1].

From a geographical point of view, Valpolicella encompasses a pedo-climatically diverse territory, within which three different terroirs are also identified, namely the larger Valpolicella Classica (north-west of the city of Verona) and Valpolicella DOC (north-east of the city of Verona), and the smaller Valpantena (north of Verona) [1]. Several recent studies have provided novel insights into the chemical characteristics of Valpolicella wines and the contribution of withering and other technological factors to their composition [2,3,4,5,6,7]. Differences in grape composition due to variations in grape area of origin at both macro- and micro-scale have been shown to induce major changes in Valpolicella wine aroma composition [4,5]. Data concerning the relevance of terroir to wines for Amarone production are however scarce, in spite of the primary commercial relevance of this product.

Among wine constituents, volatile compounds play a central role in defining wine aroma and consequently its sensorial identity. Wine aroma is the product of a biochemical and technological sequence [8,9] resulting from the contribution of different volatile molecules deriving from grapes, fermentations, and reactions linked to aging, and sometimes oak and other woods. To date, more than 800 volatile compounds such as alcohols, esters, phenols, monoterpenes, norisoprenoids, lactones, aldehydes and ketones have been identified [10,11]. The majority of wine grapes are considered non-aromatic varieties in which many of the aroma metabolites that are key to wine aroma are present in various precursor forms, with grape variety, vineyard micro-climate and training deeply affecting their occurrence [12,13]. During fermentation, yeast activity combined with acid-catalyzed reactions release some of these compounds, while other fermentation-derived volatiles such as alcohols, fatty acids and esters are also produced [14]. As a whole, under equal fermentation conditions, the resulting wine aroma would arise from the interactions between precursors and nutrient levels of the grapes (in turn related to vineyard factors) and yeast enzymatic capabilities. Different studies indicate that the levels of compounds considered to be primarily of varietal origin, such as terpenes, can also be influenced by enzymatic activities of yeasts [14,15], whereas the levels of compounds considered to be of fermentation origin, such as esters, are influenced by grape composition [7,16,17]. Accordingly, there is a generalized interest in rationalizing the relative contribution of grape origin and composition as well as of yeast strain to the expression of wine aroma composition and olfactory characteristics. Nowadays, *Saccharomyces cerevisiae* starter cultures are largely used in winemaking to limit the growth of indigenous microorganisms and achieve more predictable and desired outcomes [5,18,19,20,21,22,23,24]. Nevertheless, a growing interest in non-*Saccharomyces* yeasts and spontaneous fermentation is currently observed, primarily with the aim of exploiting their metabolic diversity and obtaining even more diversified aroma profiles [25,26].

To date, most of the studies concerning the influence of grape terroir and/or yeast strain on red wine aroma composition have been carried out on wines from non-withered grapes. However, in the context of Amarone production, the traditional practice of post-harvest withering is inducing a weight loss of approximately 30%, with major consequences for grape and wine composition. First, during withering, grape metabolism is still active and a number of metabolic changes are observed beyond the simple concentration effect due to water evaporation, including increase or decrease in the content of certain aroma compounds and precursors [27,28]. The question arises, therefore, as to whether the recently reported observations concerning the existence, in Valpolicella wines from non-withered grapes, of aroma patterns associated with grape terroir of origin [5], are still relevant in the context of a passito red wine such as Amarone. Second, the water evaporation associated with withering results in the concentration of major grape components, primarily sugars. This has major implications for yeast behavior during fermentation, affecting different enzymatic activities associated with biosynthesis of volatile compounds and increasing the relevance of metabolic phenomena such as osmotic stress [6,29,30,31].

This research paper investigated the volatile and sensory characteristics of Corvina and Corvinone wines for Amarone production from the two main terroirs of Valpolicella, in relationship to different *S. cerevisiae* strains as well as to spontaneous fermentations. The main goal was to explore the relationship between grape composition/origin and fermentation management approaches, and to unravel their respective contribution to the expression of Amarone aroma chemical and olfactive profiles. 

## 2. Materials and Methods

### 2.1. Grape Origins and Winemaking

Wines were produced with withered Corvina (*Vitis vinifera* L. cv. Corvina) or Corvinone (*Vitis vinifera*, L. cv. Corvinone) varieties. Grapes were harvested in September 2018 in vineyards belonging to the same winery and located in two terroirs within the Valpolicella appellation, namely Valpolicella DOC (Area 1) and Valpolicella Classica (Area 2). Grapes from Area 1 were obtained from three vineyard parcels located in the same estate near the town of Mezzane (45°30′36.7” N, 11°08′02.7” E). In the case of Area 2, two vineyard parcels were considered, located at a distance of approximately five kilometres from each other, in the towns of San Pietro in Cariano (45°30′40.5” N, 10°54′58.6” E) and San Giorgio in Valpolicella (45°32′16.6” N, 10°51′19.4” E), respectively. After harvesting, grapes were stored in a traditional warehouse (fruttaio) for withering. Sugar levels at harvest were in the range 195.2–207.7 g/L (Table S1). Withering lasted twelve weeks, with a gradual temperature decrease (from 16 °C to 7 °C) and a progressive increase in relative humidity (from 55% to 80%). When weight loss was approximately 30%, the grapes were pooled together in order to create two distinct vinification batches, including grapes from the parcels of Area 1 and Area 2 respectively. Grapes were manually destemmed and the berries randomized to obtain batches of 20 kg each. From each batch, eight hundred grams were taken, placed in a plastic bag and hand crushed with 80 mg of potassium metabisulphite and put into a 1.5 L glass vessel. Analytical parameters of the musts are provided in Table 1. Fermentations were carried out in duplicate with four different commercial yeasts, namely, *Saccharomyces cerevisiae* x *Saccharomyces kudriavzevi* AWRI 1503 (Yeast 1) (AB Mauri, Camellia, Australia), *Saccharomyces cerevisiae* AWRI 796 (Yeast 2) (AB Mauri, Camellia, Australia), *Saccharomyces cerevisiae* Zymaflore^®^ XPURE (Yeast 3) (Laffort, Floirac, France), and *Saccharomyces cerevisiae* Zinfandel (Yeast 4) (Vason, Verona, Italy). Active dry yeast of each commercial starter was rehydrated in water at 37 °C for 15 min, then 1.6 mL of each culture (100 g/L) was used to inoculate individual grape batches. A fifth experimental modality was also prepared, consisting of a spontaneous fermentation without the addition of potassium metabisulphite (Spontaneous). All fermentations were carried out at 22 ± 1 °C, with cap being broken twice a day by gently pressing down skins with a steel plunger, and density, weight and temperature monitored daily. Upon completion of alcoholic fermentation (glucose-fructose < 2 g/L), wines were pressed with a ten litre stainless steel basket press and supplemented with potassium metabisulphite until a final free SO_2_ concentration of 25 mg/L was achieved. Wines were then clarified by centrifugation at 4500 rpm for 15 min at 5° C (Avanti J-25, Beckman Coulter, CA, USA) and bottled in 330 mL glass bottles with crown caps, with free SO_2_ concentration of 25 mg/L.

### 2.2. Main Enological Parameters

Glucose-fructose, ammonia, primary amino nitrogen (PAN), acetic acid, and total acidity (expressed in grams of tartaric acid) were analyzed using a Biosystems Y15 multiparametric analyzer (Sinatech, Fermo, Italy). YAN (yeast assimilable nitrogen) was obtained as the sum of PAN and ammonia. For each parameter, a specific kit (Sinatech, Fermo, Italy) was used. Ethanol was analyzed with an Alcolyzer dma 4500 (Anton Paar, Graz, Austria).

### 2.3. Analysis of Volatile Compounds

For quantification of alcohols, esters, fatty acids, and benzenoids (except methyl salicylate), SPE extraction followed by GC-MS analysis was used, following the procedure described by Slaghenaufi et al. [4]. An amount of 100 µL of internal standard 2-octanol (4.2 mg/L in ethanol) was added to samples prepared with 50 mL of wine and diluted with 50 mL of deionized water. Samples were loaded onto a BOND ELUT-ENV, SPE cartridge (Agilent Technologies. Santa Clara, CA, USA) previously activated with 20 mL of dichloromethane, 20 mL of methanol and equilibrated with 20 mL of water. After sample loading, the cartridges were washed with 15 mL of water. Free volatile compounds were eluted with 10 mL of dichloromethane, and then concentrated under gentle nitrogen stream to 200 μL prior to GC injection. 

For quantification of terpenes, norisoprenoids, lactones and methyl salicylate, SPME extraction followed by GC-MS analysis was used, following the procedure described by Slaghenaufi et al. (2018) [32]. An amount of 5 µL of internal standard 2-octanol (4.2 mg/L in Ethanol) was added to 5 mL of wine diluted with 5 mL of deionized water in a 20 mL glass vial. An amount of 3 g of NaCl was added prior to GC-MS analysis. Samples were equilibrated for 1 min at 40 °C. Subsequently SPME extraction was performed using a 50/30 μm divinylbenzene–carboxen–polydimethylsiloxane (DVB/CAR/PDMS) fiber (Supelco, Bellafonte, PA, USA) exposed to sample headspace for 60 min. GC-MS analysis was carried out on an HP 7890A (Agilent Technologies) gas chromatograph coupled to a 5977B quadrupole mass spectrometer, equipped with a Gerstel MPS3 auto sampler (Müllheim/Ruhr, Germany). Separation was performed using a DB-WAX UI capillary column (30 m × 0.25, 0.25 μm film thickness, Agilent Technologies) and helium (6.0 grade) as carrier gas at 1.2 mL/min of constant flow rate. GC oven was programmed as follows: started at 40 °C for 3 min, raised to 230 °C at 4 °C/min and maintained for 20 min. Mass spectrometer was operated in electron ionization (EI) at 70 eV with ion source temperature at 250 °C and quadrupole temperature at 150 °C. Mass spectra were acquired in synchronous Scan (*m/z* 40–200) and SIM mode. Samples were analyzed in random order. 

Calibration curves were prepared for both quantification methods. For SPE-GC-MS method, a calibration curve was prepared for each analyte using seven concentration points and three replicate solutions per point in model wine (12% *v*/*v* ethanol, 3.5 g/L tartaric acid, pH 3.5) 100 µL of internal standard 2-octanol (4.2 mg/L in ethanol) was added to each calibration solution, which was then submitted to SPE extraction and GC-MS analysis as described for the samples. For SPME-GC-MS method a calibration curve was prepared for each analyte using seven concentration points and three replicate solutions per point in red wines. An amount of 5 µL of internal standards 2-octanol (4.2 mg/L in ethanol) was added to each calibration solution, which was then submitted to SPME extraction and GC-MS analysis as described for the samples.

Calibration curves were obtained using Chemstation software (Agilent Technologies, Inc.) by linear regression, plotting the response ratio (analyte peak area divided by internal standard peak area) against concentration ratio (added analyte concentration divided by internal standard concentration). Retention indices, quantitation and qualifying ions are reported in Table S2.

### 2.4. Sensory Evaluation

Sensory evaluation of the experimental wines was carried out by means of the sorting task methodology, as described by Alegre et al. (2017) [33] with slight differences. Twelve judges (6 men and 6 women), wine science researchers or teaching staff regularly involved in winemaking and/or wine evaluation participated to the sessions. They were all considered wine experts according to Parr et al. (2002) [34] specifications. One hour before the test, samples were removed from the 16 °C cold room and 20 mL was poured in ISO wine glasses (https://www.iso.org/standard/9002.html, accessed on 1 September 2021) labelled with 3-digit random codes and covered by plastic Petri dishes; all samples were served at 22 ± 1 °C, and glasses were randomized for each panelist. Panelists were asked to sort the wines into groups based on odor similarities exclusively by orthonasal evaluation, with no request to indicate specific odor descriptors. They could make as many groups as they wished.

This study contains sensory analyses carried out by a trained panel which, based on local policy, does not require the specific approval of an ethics committee.

### 2.5. Statistical Analyses 

Principal Component Analysis (PCA), Kruskal–Wallis (α = 0.05), and Hierarchical Cluster Analysis (HCA) were performed using XLSTAT 2017 (Addinsoft SARL, Paris, France). Heat map was performed with MetaboAnalyst v. 5.0 (http://www.metaboanalyst.ca, accessed on 16 March 2021), created at the University of Alberta, Edmonton, AB, Canada.

## 3. Results and Discussion

### 3.1. Main Enological Parameters

Enological parameters of withered grapes musts at crush are shown in Table 1. For each variety, Area 2 samples showed higher glucose and fructose than Area 1. pH varied across grape batches, ranging between 3.02 in Area 1 Corvinone, and 3.25 in Area 2 Corvinone. YAN content in Corvina was very similar between the two areas, while in Corvinone it was higher for Area 1.

These data reflected the natural variation in grape composition but also the effect of withering. The influence of the latter was strongly relevant in determining major differences between Areas 1 and 2, in particular for grape sugar content. While at harvest grape sugar content varied overall of less than 10 g/L (Table S1), after withering grapes of Area 2 generally exceeded those of Area 1 up to nearly 50 g/L of sugars. According to Barbanti et al. [35], withering kinetics can vary considerably depending on grape variety, bunch structure, as well as berry surface/volume ratio. In agreement with their observations, the net increase in sugar content observed here was lower in Corvinone, which is reported to have lower surface/volume ratio. Fermentation kinetics (Figure S1) showed small differences between commercial yeast strains, whereas spontaneous fermentations showed generally slower start and longer time to achieve dryness. Enological parameters of wines and their Kruskal–Wallis analysis are shown in Table 2, pH and total acidity were primarily affected by grape origins. Acetic acid was mainly influenced by yeast in both the varieties. Spontaneous fermentation showed much higher content of acetic acid than the other treatments, typically above 0.8 g/L. With an odor threshold of 0.7 g/L [36], a sensory involvement of acetic acid in several of the wines would be expected, which will be addressed later. Area 2 wines, in both varieties, in agreement with different glucose and fructose content of musts showed higher ethanol levels.

### 3.2. Volatile Compounds

The concentrations of all quantified volatile compounds, including esters, alcohols, fatty acids, terpenoids, norisoprenoids, and benzenoids, are reported in Table 3, Table 4, Table 5 and Table 6. In Corvina wines (Table 3 and Table 4), twenty-eight volatile compounds were found to be significantly different according to grape origin (α = 0.05) (Table S3). Of these, most were grape-derived compounds, such terpenes, norisoprenoids, benzenoids and C_6_ alcohols, but some fermentative compounds, were also included. Nineteen volatile compounds, mainly alcohols, esters, acids showed statistically significant differences (α = 0.05) due to yeast strain/inoculation strategy. In the case of Corvinone wines (Table 5 and Table 6), thirty-one compounds were significantly different according to grape area of origin (α = 0.05) (Table S3). In addition, many ethyl esters were impacted by grape origin. Nineteen compounds showed significant differences (α = 0.05) according to employed yeast: mainly alcohols, acids and esters.

The influence of spontaneous fermentation was assessed by comparing, for each compound, average concentrations of all inoculated fermentations with that of spontaneous fermentation (α = 0.05) (Table S4). In Corvina and Corvinone wines from Area 1, twenty-eight and twenty-one compounds were found to be significantly affected, respectively, while in Area 2 fourteen and seventeen compounds were found to be significantly affected, respectively. 

**Figure 1:**
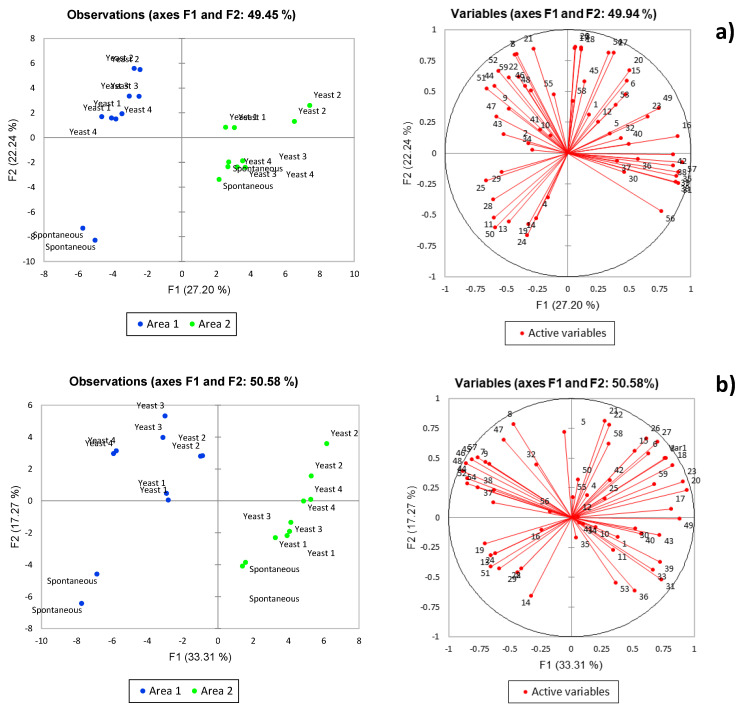
PCA of (**a**) Corvina and (**b**) Corvinone wines. Variables plot numbers correspond to: (1) 1-Butanol, (2) Butanol, (3) Isoamyl alcohol, (4) 1-Pentanol, (5) Methionol, (6) Phenylethyl alcohol, (7) 1-Hexanol, (8) *trans*-3-Hexen-1-ol, (9) *cis*-3-Hexen-1-ol, (10) *cis*-2-Hexen-1-ol, (11) Isoamyl acetate, (12) n-Hexyl acetate, (13) 2-Phenylethyl acetate, (14) Ethyl acetate, (15) Ethyl-2-methylbutanoate, (16) Ethyl 3-methylbutanoate, (17) Ethyl butanoate, (18) Ethyl hexanoate, (19) Ethyl lactate, (20) Ethyl octanoate, (21) Ethyl 3-hydroxybutanoate, (22) Ethyl 2-hydroxyhexanoate, 23) Ethyl decanoate, (24) Acetic acid, (25) 3-Methylbutanoic acid, (26) Hexanoic acid, (27) Octanoic acid, (28) *cis*-Linaloloxide, (29) *trans*-Linaloloxide, (30) 3-carene, (31) α-Phellandrene, (32) α-Terpinene, (33) β-Myrcene, (34) 1,4-Cineole, (35) Limonene, (36) 1,8-cineole, (37) p-Cymene, 38) Terpinolene, (39) Linalool, (40) Geraniol, (41) Terpinen-4-ol, (42) α-Terpineol, (43) β-Citronellol, (44) β-Damascenone, (45) TPB (E)-1-(2,3,6-Trimethylphenyl)-buta-1,3-diene), (46) TDN (1,6,-trimethyl-1,2-dihydronapthalene), (47) 3-Hydroxy-β-damascone, (48) Vitispirane, (49) Furfural, (50) Benzaldehyde, (51) Benzyl alcohol, (52) Eugenol, (53) 2,6-Dimethoxyphenol, (54) Vanillin, (55) Methyl vanillate, (56) Ethyl vanillate, (57) Methyl salicylate, (58) γ-Decalactone, (59) δ-Decalactone.

An overview of the relative influence of the different variables was obtained by Principal Component Analysis (PCA). In the case of Corvina wines (Figure 1a), 49.45% of the total variance could be explained with the first two principal components (PCs). PC1, accounting for 27.2% of the variance, was associated with grape origin, while PC2, accounting for 22.24% of the total variance, differentiated yeast strain/inoculation strategy. Separation along PC1 was mostly associated with differences in the concentration of compounds such as terpene alcohols, norisoprenoids, benzenoids and branched chain fatty acid esters, whereas along PC2, separation was mostly associated with ethyl esters, acetate esters, ethyl acetate, and higher alcohols. In the case of Corvinone wines (Figure 1b), 50.58% of the total variance was explained with the first two principal components. Additionally in this case, PC1, accounting for 33.31% of the total variance, was associated with grape origin, whereas PC2, accounting for 17.27% of the total variance, was associated with yeast strain/inoculation. Separation along PC1 was mostly associated with differences in the concentration of compounds such as terpene alcohols, norisoprenoids, benzenoids, ethyl esters, fatty acids, and higher alcohols, whereas along PC2, separation was mostly associated with ethyl acetate and methionol.

Among the compounds accounting for differences associated with grape origin, terpenes were found to be largely discriminant, with Area 2 wines showing higher content. The most abundant terpenes were β-citronellol, linalool, and in the case of Corvina, β-myrcene. The fact that terpenes were distinctive of grape origin is in agreement with other reports indicating these compounds as good markers of terroir influence [4,37,38,39,40,41]. Terpenes have also been indicated as possible varietal markers of Corvina and Corvinone wines compared with other red wines [7], so that their variations in response to grape origin appear of particular interest in the context of Valpolicella wines. Differences in wine terpene content have previously been related to the degree of grape maturity, as grape terpenes tend to increase with ripening [31,42,43]. In the present study, Area 2 had significantly higher sugar levels at crush, which appeared to be associated with an increased content of terpenes such as linalool, α-terpineol, and myrcene in the wines. It has to be considered, however, that differences in sugar content at crush were mostly due not to maturity in itself, but to withering, which can induce important modification in grape terpene content [27]. Moreover, citronellol, the most abundant among the terpene measured, showed a partially opposite behaviour, with significantly higher content in Area 1 wines for Corvina. In non-aromatic grapes such as Corvina and Corvinone, formation of compounds such as linalool is primarily due to release from glycosidic precursors [4,44], whereas citronellol mostly arises from yeast-driven reduction of the geraniol released from precursors [44]. In this sense, citronellol was also the terpene showing the largest variations due to yeast strain, supporting the view that yeast action was a main factor to the release of this compound.

Similar to terpenes, norisoprenoids were associated with different grape origins, although in this case higher levels occurred in Area 1 wines. This further confirms the lack of a clear direct association between grape sugar content and wine terpenoid content, as norisoprenoids would also be expected to increase with higher maturity. It has been previously shown that, while wine terpene content tends to decrease with withering, that of norisoprenoids tends to increase [6], so that the outcomes of withering are only partly similar to those of prolonged ripening.

Among norisoprenoids, major variations were observed for the potent odorant β-damascenone, which in Area 1 samples attained significantly higher concentrations. As in the case of citronellol, this compound arises from a complex mechanism in which grape precursor content as well as yeast ability to express both glycosidase and reductase activities are expected to play a role [45].

**Figure 2:**
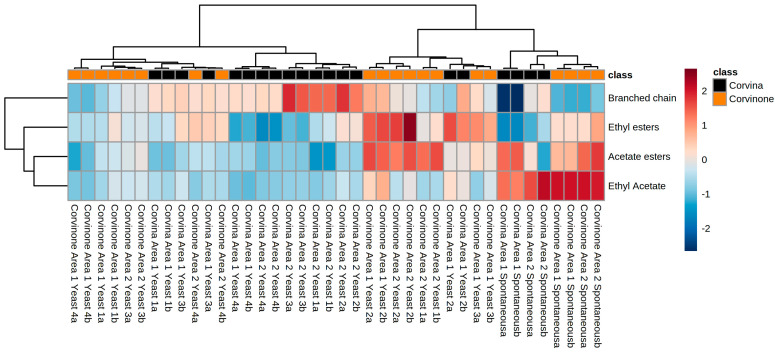
Heat map of fermentative esters in all studied wines.

Esters were another group of volatile compounds associated with significant compositional differences. In agreement with the well-known influence of yeast strain on the production of these compounds during fermentation [14], a major influence of yeast strain and spontaneous fermentation was observed (Figure 1a,b). However, depending on ester chemical class, the relationship between inoculated/spontaneous fermentation, yeast strain and ester production was also affected by grape terroir of origin, and by grape variety. This can be seen in Figure 2, a heat map with the four main esters classes analyzed in this study. 

Wines from spontaneous fermentations were well differentiated from those obtained by inoculation with commercial yeasts, regardless of grape variety and area of origin. This was due to an increased concentration of ethyl acetate and acetate esters and low content of all other esters. Most Corvinone wines were grouped together in a second cluster characterized by a higher content of ethyl and acetate esters, and this cluster also included most of the wines obtained with Yeast 2. Conversely, the majority of Corvina wines from Area 2 were in a third cluster with higher content of branched chain fatty acid esters and lower content of ethyl esters and acetates. Finally, a fourth cluster including Corvina and Corvinone wines fermented with yeasts 1, 3 and 4, were characterised by intermediate values for all the esters. 

**Figure 3:**
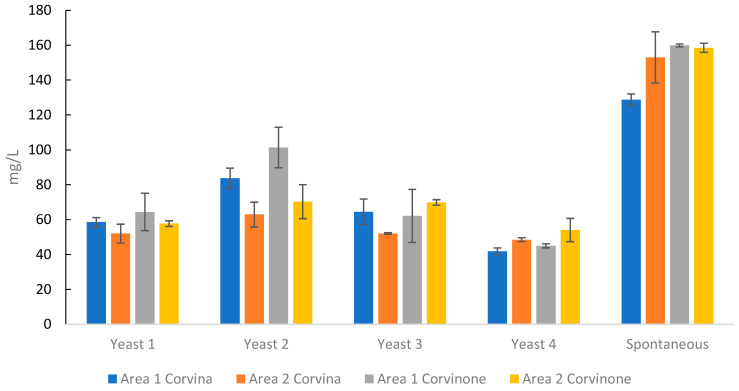
Concentration of ethyl acetate in wines obtained with different commercial strains as well as by spontaneous fermentation. Error bars indicate ± standard deviation.

Clearly, ethyl acetate production during fermentation introduced major differences in the wine ester profile. This compound is a common constituent of fermented beverages, although winemakers are commonly interested in limiting its presence due to the distinctive nail polish aroma that it can impart to wines at high concentrations. Biosynthesis of ethyl acetate in yeast is associated with the maintenance of adequate intracellular levels of CoA, which can be restored from acetyl-CoA by an appropriate alcohol acetyltransferase in the presence of ethanol, with consequent formation of ethyl acetate [46,47]. When high concentrations of both acetic acid and ethanol are present, ethyl acetate can also be formed via ethanol esterification by an esterase [46]. Spontaneous fermentations are typically associated with increased acetic acid and ethyl acetate concentrations, due to the greater proliferation of indigenous non-*Saccharomyces* species [48]. Due to their relatively low ethanol tolerance, non-*Saccharomyces* yeasts tend to proliferate in the early stages of fermentation, while with progressive accumulation of ethanol it is *S. cerevisiae* that eventually takes dominance of the fermentation medium. Therefore, it is often possible that, in the same work environment (e.g., a cellar), a given *S. cerevisiae* strain dominates both spontaneous and inoculated fermentation [48], which could have been the case also in the present study. Nevertheless, the data clearly indicated that the practice of spontaneous fermentation (which also required avoiding the addition of SO_2_) was systematically associated with specific volatile profiles that were firstly characterized by higher levels of acetic acid and ethyl acetate. As for inoculated fermentations, the data indicated that the choice of yeast strain could induce significant differences in ethyl acetate levels, which could vary up to approximately 50% for the same must, with Yeast 2 generally producing increased ethyl acetate (Figure 3). However, this same yeast was also characterized by an increased production of fatty acid ethyl esters with pleasant fruity aromas such as ethyl hexanoate and ethyl octanoate, appearing overall as a high ester producing strain.

### 3.3. Sensory Analysis

**Figure 4:**
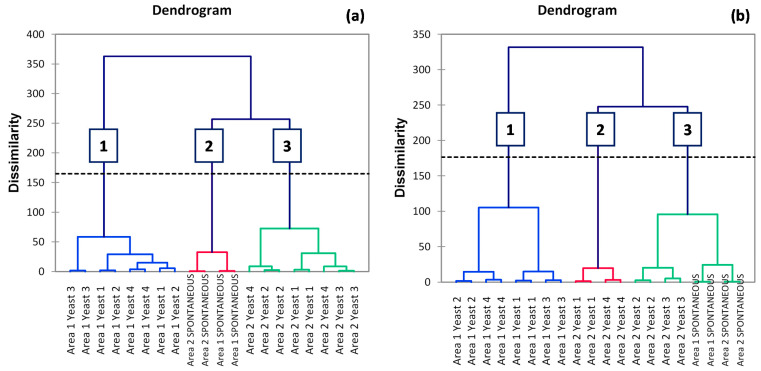
HCA of (**a**) Corvina and (**b**) Corvinone withered grapes wines sorting task data.

In studies in which multiple variables are compared, a comparative assessment of the sensory impact of individual variables can be effectively carried out evaluating the existence of odor similarity patterns that could be associated with one or more of the studied variables [49]. For this purpose, a sorting task was carried out using the samples of the study, and results elaborated by means of HCA are shown in Figure 4. Replicate wines were projected in the same group, meaning that the panel was reproducible and the wine replicates were similar. In the case of Corvina (Figure 4a), wines were clustered in three groups. A first cluster consisted of all inoculated wines from Area 1, a second cluster consisted of all the spontaneous fermentations regardless of grape origin, and finally, a third cluster consisted of all the inoculated wines from Area 2. Additionally, in the case of Corvinone (Figure 4b), three clusters were obtained. The first cluster consisted, again, of all inoculated wines from Area 1, the second cluster consisted of four wines, all from Area 2, fermented with yeast 1 and 4, and finally, the third cluster consisted of the remaining Area 2 wines and all the spontaneous fermentations. It appears therefore clear that, from a sensory perspective, not only grape origin but also the type of fermentation inoculum is strongly associated with perceivable odor differences. Indeed, taking for example the case of Corvina, on the one hand, two main clusters were observed, each containing all the samples of either Area 1 or 2 fermented with commercial strains, which confirmed the primary role of the terroir of grape origin. On the other hand, a third cluster was observed, consisting of all wines obtained by means of spontaneous fermentation, regardless of the terroir of grape origin. The contribution of yeast to the expression of terroir attributes has been investigated by different studies, and it has been often hypothesized that complex microbial consortia such as those associated with spontaneous fermentation might result in the expression of unique sensory features associated with specific vineyard sites [50]. Conversely, in the case of the present study, in particular for Corvina, it was inoculation with commercial strains that allowed differentiation based on grape origin, whereas spontaneous fermentation produced wines with distinctive odor profiles that could be clearly grouped by the panel, regardless of grape origin. 

**Figure 5:**
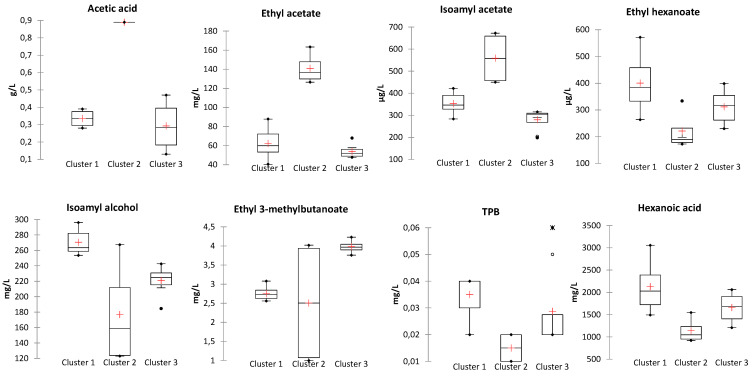
Box plots of significantly different compounds (Kruskal–Wallis, α = 0.05) between sensory clusters of Corvina wines.

**Figure 6:**
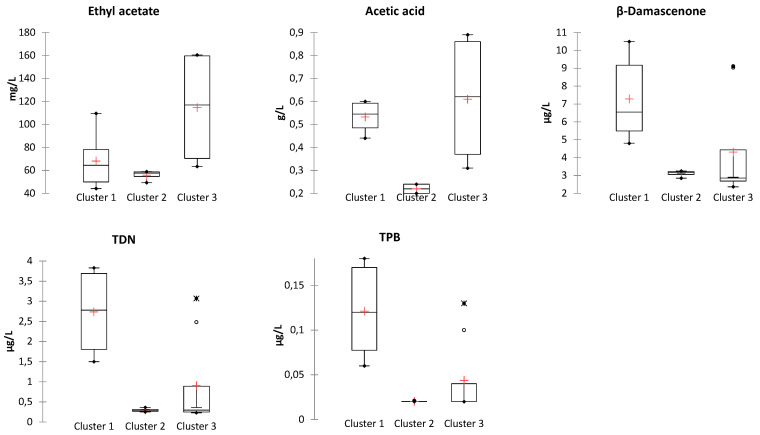
Box plots of significantly different compounds (Kruskal–Wallis, α = 0.05) between sensory clusters of Corvinone wines.

In order to identify the volatile compounds most likely contributing to the observed clusters, sorting task data were compared with wines’ volatile composition. In the case of Corvina, thirty-five volatile compounds showed a significantly different content between clusters, thirteen in the case of Corvinone (Table S5). Among these, a comparison of compounds with an odor activity value (OAV) higher than one, which are expected to contribute more prominently to wine aroma, was carried out (Figure 5 and Figure 6). 

The data obtained indicated that, both for Corvina and Corvinone, the cluster associated with spontaneous fermentation (namely clusters 2 and 3, respectively) was characterized by significantly higher levels of acetic acid and ethyl acetate. This appears in agreement with the observation that the initial stages of spontaneous fermentation are typically associated with increased proliferation of non-*Saccharomyces* yeasts, some of which are characterized by high production of ethyl acetate and acetic acid [51,52]. Additionally, the particularly high levels of these compounds may also be due to a response to osmotic stress [29] from the high glucose and fructose content (up to almost 300 g/L) following the withering treatment. Ethyl acetate is often described as having a nail lacquer odor, whereas acetic acid is the main odor compound of vinegar. These compounds are likely to contribute to the specific odor character of wines from spontaneous fermentation, also in combination with the fact that the levels of other compounds such as norisoprenoids and ethyl esters were generally lower in samples from spontaneous fermentations. 

Further interesting insights were obtained concerning the contribution of other aroma compounds to the odor profile of the other clusters observed. In the case of Corvina (Figure 5), wines from cluster 1, all inoculated from Area 1, compared with cluster 3, all inoculated from Area 2, showed a higher content of ethyl butanoate and ethyl hexanoate (fruity), TPB ((E)-1-(2,3,6-Trimethylphenyl)-buta-1,3-diene)(tobacco), TDN (1,6,-trimethyl-1,2-dihydronapthalene) (kerosene), β-damascenone (quince), eugenol (cloves) and hexanoic acid (rancid). Concerning Corvinone, wines from cluster 1 all inoculated from Area 1, showed higher levels of norisoprenoids and eugenol (Figure 6), whereas wines of cluster 2 (Area 2 yeast 1 and 4) were characterized by low levels of acetic acid, below the odor threshold. Although these compounds were present in concentrations exceeding their odor threshold, at the levels at which they were detected they are not expected to specifically impart their odor to the wines of each cluster, but mostly to act synergistically to determine the overall sensory nuances perceived during tasting [11]. 

## 4. Conclusions

In conclusion, most aroma-related volatile metabolites of Corvina and Corvinone wines from withered grapes were affected by grape terroir of origin, highlighting the central role of grape selection for obtaining defined Amarone aroma profiles. Sensory analysis confirmed this observation, since grape origin was the major driver of the sensory differences observed. Nevertheless, when spontaneous fermentation was applied, wine sensory characteristics associated with either grape terroir or variety were less prominent, and this was due to increased levels of ethyl acetate and acetic acid. Other volatile compounds, in particular, norisoprenoids and ethyl esters, contributed to expressing sensory diversity associated with either yeast strain or grape terroir of origin. Additional sensory work in determining the actual contribution of these compounds to Amarone aroma is needed. 

The results of this study represent a first attempt to classify the relative contribution of different industry-relevant variables to the management of the Amarone aroma profile. Further work is needed to address more specifically the technological factors determining some of the behaviours observed, in particular, concerning yeast ethyl acetate metabolism. This will enable winemakers to rationalize production choices towards specific sensory quality objectives. 

Limitation of the study: Equipment: The laboratory did not have a sensory room equipped with individual boots. Time and method: Identification of the bacterial population in spontaneous fermentations would have been interesting information, even if not indispensable to the study.

## Figures and Tables

**Table 1:** Enological parameters of musts at crush.

	Glucose and Fructose (g/L)	pH	PAN ^1^ (mg/L)	AMMONIA (mg/L)	YAN ^2^ (mg/L)
	Mean ± sd	Mean ± sd	Mean ± sd	Mean ± sd	Mean ± sd
Area 1 Corvina	243.8 ± 3.1c	3.17 ± 0.03c	111.9 ± 7.8c	36.8 ± 2.4c	142.2 ± 9.3c
Area 2 Corvina	291.2 ± 3.6a	3.36 ± 0.04a	105.0 ± 5.4c	46.3 ± 6.2b	143.1 ± 6.4c
Area 1 Corvinone	235.1 ± 2.4d	3.02 ± 0.01d	149.3 ± 8.4a	73.9 ± 4.4a	210.1 ± 11.3a
Area 2 Corvinone	254.9 ± 4.2b	3.25 ± 0.01b	124.1 ± 9.1b	49.9 ± 3.6b	165.1 ± 11.8b

^1^ PAN: primary amino nitrogen; ^2^ YAN: yeast assimilable nitrogen. Different letters in the same column denote statistically significant difference as obtained by Kruskal–Wallis (α = 0.05) with Dunn multiple pairwise comparison.

**Table 2:** Enological parameters of wines at the end of alcoholic fermentation (glucose and fructose <2 g/L).

		Yeast 1	Yeast 2	Yeast 3	Yeast 4	Spontaneous
		mean ± sd	mean ± sd	mean ± sd	mean ± sd	mean ± sd
Area 1 Corvina	Total acidity (g/L of tartaric acid)	6.7 ± 0.1b	7.7 ± 0.1a	6.7 ± 0.1b	7.4 ± 0.1a	7.8 ± 1.9a
	pH	3.01 ± 0.01b	3.08 ± 0.01a	3.07 ± 0.01a	3.07 ± 0.01a	3.09 ± 0.02a
	Acetic acid (g/L)	0.28 ± 0.02c	0.30 ± 0.00c	0.37 ± 0.04b	0.39 ± 0.01b	0.89 ± 0.01a
	Ethanol (% *v*/*v*)	14.77 ± 0.31a	14.69 ± 0.09a	14.82 ± 0.42a	14.66 ± 0.31a	14.65 ± 0.30a
Area 2 Corvina	Total acidity (g/L of tartaric acid)	7.6 ± 0.7b	10.3 ± 0.3a	7.8 ± 0.1b	7.6 ± 0.2b	7.8 ± 0b
	pH	2.98 ± 0.01a	2.99 ± 0.01a	2.98 ± 0.02a	2.98 ± 0.00a	2.92 ± 0.01b
	Acetic acid (g/L)	0.20 ± 0.11c	0.47 ± 0.18b	0.37 ± 0.18b	0.13 ± 0.01c	0.89 ± 0.06a
	Ethanol (% *v*/*v*)	17.91 ± 0.40a	17.73 ± 0.09a	17.86 ± 0.11a	17.68 ± 0.08a	17.41 ± 0.24a
Area 1 Corvinone	Total acidity (g/L of tartaric acid)	5.8 ± 0.1c	7.2 ± 0.2a	6.4 ± 0b	6 ± 0b	6.2 ± 0.3b
	pH	3.35 ± 0.06a	3.30 ± 0.01a	3.32 ± 0.01a	3.31 ± 0.02a	3.23 ± 0.01b
	Acetic acid (g/L)	0.50 ± 0.04c	0.60 ± 0.07b	0.59 ± 0.01b	0.44 ± 0.03d	0.85 ± 0.01a
	Ethanol (% *v*/*v*)	14.34 ± 0.17a	14.17 ± 0.35a	14.24 ± 0.31a	14.54 ± 0.09a	14.16 ± 0.02a
Area 2 Corvinone	Total acidity (g/L of tartaric acid)	6.6 ± 0.37b	7.81 ± 0.1a	6.62 ± 0.1b	7.04 ± 0.0b	6.84 ± 0.4b
	pH	3.21 ± 0.01a	3.22 ± 0.01a	3.22 ± 0.01a	3.23 ± 0.01a	3.21 ± 0.0a
	Acetic acid (g/L)	0.23 ± 0.01c	0.41 ± 0.21b	0.28 ± 0.02b	0.21 ± 0.02c	0.82 ± 0.01a
	Ethanol (% *v*/*v*)	15.50 ± 0.12a	15.41 ± 0.03a	15.31 ± 0.41a	15.32 ± 0.21a	15.51 ± 0.5a

Different letters in the same row denote statistically significant difference as obtained by Kruskal–Wallis (α = 0.05) with Dunn multiple pairwise comparison.

**Table 3:** Concentration (µg/L) of volatile compounds of Area 1 Corvina wines.

	Yeast 1	Yeast 2	Yeast 3	Yeast 4	Spontaneous
	mean ± sd	mean ± sd	mean ± sd	mean ± sd	mean ± sd
**Alcohols**					
1-Butanol	371.05 ± 14.63	241.87 ± 9.91	186.51 ± 6.10	241.95 ± 3.30	146.94 ± 2.74
2-Butanol	4090.42 ± 127.27	4366.08 ± 114.18	7199.53 ± 88.06	4483.68 ± 86.56	4814.56 ± 106.43
1-Pentanol	41.94 ± 2.13	44.76 ± 0.83	46.75 ± 2.60	46.35 ± 3.87	52.11 ± 0.41
Isoamyl alcohol (mg/L)	260.1 ± 5.16	270.95 ± 10.67	294.79 ± 1.93	256.68 ± 4.26	123.5 ± 0.57
Phenylethyl alcohol (mg/L)	24.75 ± 0.92	17.69 ± 0.4	20.82 ± 0.61	23.56 ± 2.11	9.50 ± 0.15
Methionol	105.27 ± 4.96	84.92 ± 5.44	123.47 ± 14.62	143.71 ± 3.83	33.01 ± 1.29
**C_6_ alcohols**					
1-Hexanol	954.18 ± 9.50	948.77 ± 8.58	902.40 ± 8.26	853.42 ± 15.13	768.00 ± 24.98
*trans*-3-Hexen-1-ol	9.30 ± 0.02	9.92 ± 0.94	10.55 ± 0.59	11.07 ± 0.26	6.79 ± 0.35
*cis*-3-Hexen-1-ol	43.27 ± 1.94	40.99 ± 0.93	44.51 ± 1.95	39.98 ± 1.09	40.64 ± 1.39
*cis*-2-Hexen-1-ol	18.26 ± 0.17	15.85 ± 0.70	16.41 ± 1.18	16.84 ± 0.79	16.23 ± 1.92
**Acetate esters**					
Isoamyl acetate	287.14 ± 4.84	417.00 ± 6.83	365.18 ± 25.42	342.91 ± 3.76	663.35 ± 12.79
n-Hexyl acetate	19.11 ± 0.08	51.72 ± 2.94	18.96 ± 0.12	17.36 ± 0.36	23.57 ± 1.29
2-Phenylethyl acetate	23.91 ± 2.09	18.75 ± 0.72	19.87 ± 0.37	23.83 ± 2.04	44.81 ± 0.71
Ethyl acetate (mg/L)	58.49 ± 2.7	83.66 ± 5.86	64.52 ± 7.29	41.86 ± 1.93	128.73 ± 3.26
**Branched-chain fatty acids ethyl esters**					
Ethyl 2-methylbutanoate	3.41 ± 0.12	2.73 ± 1.39	3.38 ± 0.01	3.17 ± 0.23	0.90 ± 0.14
Ethyl 3-methylbutanoate	2.57 ± 0.01	2.95 ± 0.19	2.73 ± 0.08	2.79 ± 0.21	1.05 ± 0.07
**Fatty acids ethyl esters**					
Ethyl butanoate	260.10 ± 29.42	422.74 ± 22.32	251.05 ± 11.19	184.12 ± 0.15	95.45 ± 1.34
Ethyl hexanoate	361.31 ± 15.52	555.12 ± 23.57	414.17 ± 24.71	271.97 ± 11.41	176.53 ± 5.61
Ethyl octanoate	171.19 ± 15.40	309.87 ± 24.84	247.56 ± 2.04	148.21 ± 7.71	89.70 ± 3.05
Ethyl lactate	225.35 ± 7.00	236.10 ± 20.09	284.54 ± 2.35	203.32 ± 12.91	359.76 ± 0.62
Ethyl decanoate	65.28 ± 5.73	42.70 ± 1.45	45.31 ± 2.35	14.25 ± 1.29	17.94 ± 0.08
**Other esters**					
Ethyl 3-hydroxybutanoate	148.56 ± 7.57	247.18 ± 15.42	227.89 ± 12.49	139.87 ± 6.30	78.60 ± 2.91
Ethyl 2-hydroxyhexanoate	0.42 ± 0.02	1.23 ± 0.16	1.52 ± 0.10	1.01 ± 0.00	0.53 ± 0.01
**Fatty acids**					
3-Methylbutanoic acid	424.86 ± 19.17	310.26 ± 9.60	439.13 ± 20.92	414.41 ± 11.50	547.27 ± 5.76
Hexanoic acid	1824.83 ± 50.37	2973.50 ± 117.13	2209.76 ± 14.81	1509.15 ± 23.80	941.09 ± 29.54
Octanoic acid	3537.02 ± 193.32	4631.18 ± 308.33	4100.89 ± 2.94	3219.52 ± 27.57	2216.20 ± 164.18
**Terpenoids**					
*cis*-Linaloloxide	0.55 ± 0.05	0.10 ± 0.01	0.08 ± 0.02	0.02 ± 0.00	0.68 ± 0.03
*trans*-Linaloloxide	0.96 ± 0.07	0.03 ± 0.01	0.05 ± 0.03	0.16 ± 0.13	0.62 ± 0.04
Linalool	5.76 ± 1.00	6.98 ± 0.42	5.81 ± 0.36	5.07 ± 0.13	5.06 ± 0.10
Geraniol	2.23 ± 0.13	3.50 ± 0.69	3.35 ± 0.62	2.67 ± 0.14	3.01 ± 0.32
α-Terpineol	3.50 ± 0.00	3.90 ± 0.52	2.82 ± 0.33	3.22 ± 0.13	2.81 ± 0.40
β-Citronellol	26.76 ± 3.07	8.57 ± 1.70	13.30 ± 1.49	16.31 ± 0.43	15.69 ± 1.77
α-Phellandrene	3.35 ± 0.27	3.66 ± 0.16	3.16 ± 0.06	1.26 ± 0.06	3.20 ± 0.14
α-Terpinen	0.13 ± 0.01	0.16 ± 0.01	0.11 ± 0.01	0.45 ± 0.07	0.14 ± 0.00
β-Myrcene	4.36 ± 0.35	5.65 ± 0.35	3.89 ± 0.23	2.83 ± 0.04	4.80 ± 1.10
Limonene	0.74 ± 0.04	0.93 ± 0.08	0.63 ± 0.04	0.67 ± 0.02	0.78 ± 0.16
1,4-Cineole	nd	nd	0.04 ± 0.00	0.43 ± 0.03	0.09 ± 0.01
1,8-Cineole	0.08 ± 0.00	0.04 ± 0.06	0.13 ± 0.01	0.05 ± 0.01	0.08 ± 0.01
p-Cymene	0.32 ± 0.01	0.39 ± 0.01	0.28 ± 0.02	0.52 ± 0.02	0.39 ± 0.01
Terpinolene	0.47 ± 0.08	0.59 ± 0.02	0.45 ± 0.07	0.52 ± 0.01	0.52 ± 0.04
Terpinen-4-ol	0.48 ± 0.04	0.47 ± 0.09	1.21 ± 0.15	14.11 ± 0.01	0.75 ± 0.07
**Norisoprenoids**					
β-Damascenone	8.33 ± 1.56	8.38 ± 1.17	5.09 ± 0.30	6.87 ± 0.16	6.01 ± 0.81
3-Hydroxy-β-damascone	0.21 ± 0.01	0.40 ± 0.04	0.28 ± 0.09	0.36 ± 0.04	0.30 ± 0.01
Vitispirane	3.45 ± 0.21	3.88 ± 0.18	2.66 ± 0.04	3.62 ± 0.02	3.10 ± 0.99
TPB	0.04 ± 0.01	0.04 ± 0.00	0.03 ± 0.01	0.04 ± 0.00	0.02 ± 0.00
TDN	0.73 ± 0.03	0.78 ± 0.13	0.50 ± 0.06	0.69 ± 0.03	0.57 ± 0.07
**Benzenoids and others**					
Benzyl alcohol	279.52 ± 13.31	296.78 ± 13.09	316.75 ± 7.83	307.61 ± 26.40	251.22 ± 11.33
Vanillin	5.50 ± 0.00	5.52 ± 0.12	5.43 ± 0.15	5.54 ± 0.16	2.00 ± 0.07
Ethyl vanillate	127.88 ± 5.50	119.02 ± 3.81	123.33 ± 3.86	124.45 ± 2.86	126.81 ± 5.37
Methyl vanillate	5.46 ± 0.20	8.72 ± 0.63	5.17 ± 0.76	6.23 ± 0.23	5.49 ± 0.52
Benzaldehyde	16.14 ± 1.19	20.31 ± 1.63	16.66 ± 0.59	17.85 ± 0.70	70.95 ± 7.71
Eugenol	7.01 ± 0.04	7.32 ± 0.02	7.26 ± 0.00	7.14 ± 0.22	6.45 ± 0.49
Methyl salicylate	0.99 ± 0.16	1.12 ± 0.31	0.63 ± 0.01	0.74 ± 0.05	0.39 ± 0.07
2,6-Dimethoxyphenol	5.30 ± 0.55	5.42 ± 0.37	6.29 ± 0.16	6.39 ± 0.18	5.02 ± 0.16
Furfural	1.38 ± 0.06	1.24 ± 0.09	1.89 ± 0.00	1.17 ± 0.18	0.69 ± 0.04
γ-Decalactone	2.36 ± 0.16	2.41 ± 0.13	2.27 ± 0.00	2.73 ± 0.01	2.15 ± 0.19
δ-Decalactone	32.99 ± 2.51	31.19 ± 1.33	28.78 ± 1.27	30.24 ± 3.59	23.71 ± 2.13

Nd means not detected. TPB is short for (E)-1-(2,3,6-Trimethylphenyl)-buta-1,3-diene. TDN is short for 1,6,-trimethyl-1,2-dihydronapthalene.

**Table 4:** Concentration (µg/L) and standard deviation (± µg/L) of volatile compounds of Area 2 Corvina wines.

	Yeast 1	Yeast 2	Yeast 3	Yeast 4	Spontaneous
	mean ± sd	mean ± sd	mean ± sd	mean ± sd	mean ± sd
**Alcohols**					
1-Butanol	441.60 ± 12.31	220.49 ± 9.52	190.27 ± 8.34	205.29 ± 9.91	290.30 ± 14.21
2-Butanol	3487.83 ± 19.06	4174.76 ± 194.86	4787.57 ± 1.14	3691.44 ± 374.75	5230.78 ± 139.41
1-Pentanol	36.54 ± 3.17	46.36 ± 2.33	47.77 ± 6.75	43.88 ± 3.17	53.30 ± 16.89
Isoamyl alcohol (mg/L)	224.67 ± 6.78	229.71 ± 18.30	223.20 ± 16.50	207.07 ± 31.67	230.41 ± 52.37
Phenylethyl alcohol (mg/L)	23.78 ± 0.78	21.43 ± 0.24	21.91 ± 0.45	21.16 ± 0.71	22.77 ± 0.71
Methionol	102.96 ± 3.90	98.07 ± 9.43	127.90 ± 5.51	153.40 ± 17.46	162.44 ± 107.20
**C_6_ alcohols**					
1-Hexanol	838.43 ± 25.46	781.33 ± 10.08	712.71 ± 4.66	798.18 ± 16.11	763.07 ± 26.74
*trans*-3-Hexen-1-ol	7.30 ± 0.41	7.90 ± 0.24	6.53 ± 0.43	7.31 ± 0.42	6.82 ± 1.25
*cis*-3-Hexen-1-ol	38.18 ± 3.79	39.27 ± 1.43	34.32 ± 0.60	38.98 ± 1.32	39.13 ± 4.83
*cis*-2-Hexen-1-ol	16.33 ± 1.27	16.70 ± 0.50	15.82 ± 0.24	16.70 ± 0.08	16.24 ± 0.14
**Acetate esters**					
Isoamyl acetate	201.22 ± 3.98	310.27 ± 1.60	305.31 ± 3.94	302.26 ± 18.75	342.06 ± 165.76
n-Hexyl acetate	20.18 ± 1.35	38.97 ± 1.15	22.50 ± 2.48	22.36 ± 1.57	32.38 ± 3.58
2-Phenylethyl acetate	22.27 ± 0.41	25.38 ± 1.02	22.87 ± 1.31	14.91 ± 2.82	19.87 ± 0.33
Ethyl acetate (mg/L)	51.97 ± 5.39	62.89 ± 7.08	52.04 ± 0.48	48.51 ± 1.12	153.00 ± 14.63
**Branched-chain fatty acids ethyl esters**					
Ethyl 2-methylbutanoate	3.50 ± 0.09	3.86 ± 0.68	3.99 ± 0.11	2.22 ± 0.13	1.80 ± 0.33
Ethyl 3-methylbutanoate	4.07 ± 0.08	3.96 ± 0.09	4.08 ± 0.21	3.83 ± 0.10	3.97 ± 0.08
**Fatty acids ethyl esters**					
Ethyl butanoate	232.99 ± 3.66	244.30 ± 3.06	170.31 ± 6.74	160.74 ± 7.11	172.36 ± 21.39
Ethyl hexanoate	342.78 ± 2.55	391.36 ± 10.20	280.57 ± 14.14	232.48 ± 3.34	265.86 ± 95.90
Ethyl octanoate	232.65 ± 16.50	296.96 ± 3.34	201.46 ± 6.02	152.80 ± 1.60	182.44 ± 49.89
Ethyl lactate	244.39 ± 18.96	246.63 ± 7.59	236.89 ± 3.22	193.08 ± 7.44	336.42 ± 28.96
Ethyl decanoate	70.89 ± 1.82	82.04 ± 7.63	56.46 ± 3.37	29.78 ± 6.25	36.49 ± 4.51
**Other esters**					
Ethyl 3-hydroxybutanoate	105.64 ± 6.74	134.61 ± 0.52	65.35 ± 0.66	77.05 ± 16.40	83.53 ± 2.35
Ethyl 2-hydroxyhexanoate	0.25 ± 0.02	0.54 ± 0.06	0.43 ± 0.00	0.46 ± 0.04	0.36 ± 0.24
**Fatty acids**					
3-Methylbutanoic acid	318.40 ± 7.15	328.26 ± 33.71	388.00 ± 3.51	319.32 ± 21.51	269.95 ± 123.81
Hexanoic acid	2030.04 ± 44.08	1844.35 ± 46.25	1451.38 ± 138.40	1316.03 ± 152.40	1338.87 ± 293.02
Octanoic acid	4261.98 ± 68.70	3990.88 ± 62.36	3355.84 ± 49.80	3190.36 ± 183.16	3272.96 ± 553.04
**Terpenoids**					
*cis*-Linaloloxide	0.09 ± 0.01	0.09 ± 0.03	0.07 ± 0.04	0.04 ± 0.01	0.06 ± 0.01
*trans*-Linaloloxide	0.24 ± 0.01	0.06 ± 0.02	0.05 ± 0.01	0.03 ± 0.00	0.09 ± 0.00
Linalool	6.45 ± 0.63	12.31 ± 0.06	10.42 ± 0.35	10.16 ± 0.02	8.81 ± 0.21
Geraniol	2.81 ± 0.11	4.25 ± 0.16	3.03 ± 0.06	3.21 ± 0.21	3.02 ± 0.49
α-Terpineol	5.21 ± 0.01	10.13 ± 0.52	6.46 ± 0.33	4.42 ± 0.26	3.98 ± 0.16
β-Citronellol	14.28 ± 0.25	10.02 ± 0.18	9.40 ± 0.81	9.18 ± 0.39	9.02 ± 1.48
α-Phellandrene	5.70 ± 0.23	10.37 ± 0.98	5.92 ± 0.57	6.62 ± 0.26	6.70 ± 0.28
α-Terpinen	0.20 ± 0.00	0.68 ± 0.54	0.20 ± 0.00	0.19 ± 0.01	0.20 ± 0.01
β-Myrcene	7.31 ± 0.43	13.46 ± 1.27	7.69 ± 0.74	8.59 ± 0.34	8.70 ± 0.36
Limonene	1.38 ± 0.05	2.51 ± 0.14	1.36 ± 0.11	1.46 ± 0.10	1.26 ± 0.02
1,4-Cineole	0.00 ± 0.00	0.05 ± 0.00	0.08 ± 0.00	0.06 ± 0.01	0.06 ± 0.00
1,8-Cineole	0.12 ± 0.02	0.19 ± 0.01	0.09 ± 0.00	0.09 ± 0.01	0.08 ± 0.01
p-Cymene	0.39 ± 0.04	0.55 ± 0.01	0.45 ± 0.03	0.40 ± 0.03	0.34 ± 0.10
Terpinolene	0.84 ± 0.05	1.64 ± 0.23	0.84 ± 0.05	0.85 ± 0.04	0.75 ± 0.05
Terpinen-4-ol	1.23 ± 0.04	1.17 ± 0.06	2.44 ± 0.20	1.59 ± 0.18	1.16 ± 1.12
**Norisoprenoids**					
β-Damascenone	3.34 ± 0.13	5.00 ± 0.26	2.81 ± 0.16	2.89 ± 0.13	2.23 ± 0.20
3-Hydroxy-β-damascone	0.31 ± 0.04	0.13 ± 0.02	0.16 ± 0.01	0.16 ± 0.01	0.29 ± 0.08
Vitispirane	1.83 ± 0.08	4.01 ± 0.18	1.59 ± 0.07	1.56 ± 0.12	0.76 ± 0.21
TPB	0.02 ± 0.00	0.06 ± 0.01	0.02 ± 0.00	0.02 ± 0.00	0.01 ± 0.00
TDN	0.32 ± 0.02	0.75 ± 0.06	0.24 ± 0.03	0.23 ± 0.03	0.13 ± 0.00
**Benzenoids and others**					
Benzyl alcohol	199.41 ± 1.32	223.31 ± 21.96	193.43 ± 0.15	233.75 ± 2.73	256.88 ± 5.20
Vanillin	4.97 ± 0.02	4.97 ± 0.06	4.90 ± 0.19	4.87 ± 0.15	4.88 ± 0.14
Ethyl vanillate	135.24 ± 4.24	143.78 ± 4.97	137.46 ± 5.40	140.60 ± 7.06	143.45 ± 4.02
Methyl vanillate	4.71 ± 0.10	6.05 ± 0.60	5.92 ± 0.39	6.00 ± 0.80	4.39 ± 0.20
Benzaldehyde	15.42 ± 0.74	15.45 ± 0.39	15.60 ± 0.86	16.59 ± 1.25	15.47 ± 1.19
Eugenol	6.55 ± 0.35	6.28 ± 0.01	5.46 ± 0.01	5.14 ± 0.17	6.00 ± 0.02
Methyl salicylate	3.64 ± 0.07	5.73 ± 0.09	2.32 ± 0.05	2.99 ± 0.08	2.35 ± 0.17
2,6-Dimethoxyphenol	6.51 ± 0.01	6.57 ± 0.69	5.51 ± 0.16	5.30 ± 0.45	5.78 ± 0.89
Furfural	1.68 ± 0.11	2.06 ± 0.35	1.54 ± 0.08	1.43 ± 0.12	1.53 ± 0.13
γ-Decalactone	2.60 ± 0.14	2.25 ± 0.01	2.36 ± 0.15	2.44 ± 0.07	2.26 ± 0.01
δ-Decalactone	25.14 ± 8.63	21.78 ± 2.94	25.17 ± 3.28	23.91 ± 0.01	23.58 ± 1.71

Nd means not detected. TPB is short for (E)-1-(2,3,6-Trimethylphenyl)-buta-1,3-diene. TDN is short for 1,6,-trimethyl-1,2-dihydronapthalene.

**Table 5:** Concentration (µg/L) of volatile compounds of Area 1 Corvinone withered wines.

	Yeast 1	Yeast 2	Yeast 3	Yeast 4	Spontaneous
	mean ± sd	mean ± sd	mean ± sd	mean ± sd	mean ± sd
**Alcohols**					
1-Butanol	326.74 ± 172.01	228.80 ± 28.38	158.29 ± 15.66	100.38 ± 4.53	141.27 ± 17.97
2-Butanol	5643.65 ± 610.53	4982.00 ± 428.99	5859.68 ± 54.54	3285.67 ± 159.30	10321.02 ± 654.72
1-Pentanol	48.14 ± 0.59	43.09 ± 3.20	46.67 ± 7.15	59.99 ± 4.31	42.16 ± 1.04
Isoamyl alcohol (mg/L)	254.28 ± 83.86	27.14 ± 10.10	28.44 ± 27.64	200.64 ± 13.64	179.62 ± 7.03
Phenylethyl alcohol (mg/L)	21.33 ± 0.99	19.42 ± 0.03	19.27 ± 2.09	20.95 ± 0.99	14671 ± 1.35
Methionol	221.98 ± 5.51	135.32 ± 28.73	222.60 ± 31.58	389.66 ± 73.67	51.24 ± 15.38
**C_6_ alcohols**					
1-Hexanol	2020.95 ± 110.53	1940.31 ± 3.38	1737.24 ± 135.60	1701.42 ± 125.04	1710.03 ± 34.29
*trans*-3-Hexen-1-ol	27.90 ± 1.53	27.90 ± 0.91	29.85 ± 0.43	25.66 ± 1.57	17.26 ± 3.01
*cis*-3-Hexen-1-ol	17.83 ± 0.05	20.29 ± 1.92	19.81 ± 1.68	18.76 ± 1.36	19.12 ± 4.82
*cis*-2-Hexen-1-ol	16.54 ± 0.03	15.05 ± 0.83	14.73 ± 0.55	16.21 ± 0.88	15.78 ± 1.07
**Acetate esters**					
Isoamyl acetate	382.14 ± 9.68	707.32 ± 20.52	482.77 ± 29.00	258.90 ± 31.35	547.90 ± 4.36
n-Hexyl acetate	17.77 ± 0.84	14.17 ± 1.07	8.98 ± 0.74	12.35 ± 2.60	11.60 ± 0.49
2-Phenylethyl acetate	35.58 ± 0.69	29.21 ± 0.07	25.74 ± 1.08	24.61 ± 1.47	42.36 ± 1.78
Ethyl acetate (mg/L)	64.37 ± 10.73	101.32 ± 11.63	62.12 ± 15.22	44.99 ± 1.17	159.83 ± 0.86
**Branched-chain fatty acids ethyl esters**					
Ethyl 2-methylbutanoate	1.80 ± 0.35	3.57 ± 0.21	2.46 ± 0.31	1.21 ± 0.21	0.89 ± 0.16
Ethyl 3-methylbutanoate	3.13 ± 0.01	3.10 ± 0.16	3.22 ± 0.13	3.06 ± 0.09	3.19 ± 0.10
**Fatty acids ethyl esters**					
Ethyl butanoate	159.71 ± 5.76	206.20 ± 1.07	184.08 ± 12.60	168.03 ± 9.72	125.35 ± 16.24
Ethyl hexanoate	288.88 ± 34.73	441.20 ± 3.88	371.86 ± 22.02	294.37 ± 1.03	136.72 ± 21.62
Ethyl octanoate	130.27 ± 11.62	204.82 ± 17.71	182.78 ± 16.91	117.46 ± 7.09	63.44 ± 3.55
Ethyl lactate	467.73 ± 59.32	685.54 ± 32.99	502.66 ± 4.44	415.45 ± 8.14	1047.19 ± 10.42
Ethyl decanoate	31.85 ± 2.35	45.66 ± 2.81	54.99 ± 2.70	29.99 ± 3.01	6.92 ± 1.84
**Other esters**					
Ethyl 3-hydroxybutanoate	219.48 ± 14.79	291.85 ± 20.39	373.57 ± 19.01	169.63 ± 3.90	36.88 ± 0.49
Ethyl 2-hydroxyhexanoate	0.40 ± 0.10	1.36 ± 0.03	1.22 ± 0.02	0.66 ± 0.03	0.22 ± 0.09
**Fatty acids**					
3-Methylbutanoic acid	323.93 ± 49.67	364.47 ± 17.78	486.21 ± 29.49	253.57 ± 14.10	363.79 ± 279.85
Hexanoic acid	1882.76 ± 89.07	2938.93 ± 68.25	2359.40 ± 124.09	1986.87 ± 33.29	1022.20 ± 80.26
Octanoic acid	3576.79 ± 173.28	4432.38 ± 27.19	4197.55 ± 25.65	3798.66 ± 22.14	1735.40 ± 831.93
**Terpenoids**					
*cis*-Linaloloxide	0.11 ± 0.08	0.06 ± 0.02	0.08 ± 0.04	0.05 ± 0.02	0.95 ± 1.24
*trans*-Linaloloxide	0.07 ± 0.01	0.03 ± 0.01	0.08 ± 0.08	0.04 ± 0.01	0.32 ± 0.26
Linalool	4.36 ± 0.19	5.06 ± 0.50	4.49 ± 0.30	1.55 ± 0.07	5.05 ± 0.25
Geraniol	0.83 ± 0.25	1.14 ± 0.26	1.15 ± 0.21	1.32 ± 0.17	1.10 ± 0.17
α-Terpineol	3.19 ± 0.57	3.57 ± 0.53	4.06 ± 0.67	3.37 ± 0.04	3.39 ± 0.11
β-Citronellol	5.62 ± 1.79	4.58 ± 0.16	7.25 ± 0.38	3.68 ± 0.04	3.53 ± 0.64
α-Phellandrene	1.08 ± 0.01	1.38 ± 0.07	1.74 ± 0.17	0.41 ± 0.01	2.19 ± 0.42
α-Terpinen	0.06 ± 0.01	0.09 ± 0.01	0.09 ± 0.02	0.22 ± 0.02	0.10 ± 0.01
β-Myrcene	1.40 ± 0.03	1.80 ± 0.09	2.26 ± 0.23	2.64 ± 0.11	2.85 ± 0.54
Limonene	0.40 ± 0.01	0.46 ± 0.02	0.61 ± 0.11	0.85 ± 0.04	0.69 ± 0.08
1,4-Cineole	0.03 ± 0.01	0.02 ± 0.00	nd	nd	nd
1,8-Cineole	0.09 ± 0.01	0.08 ± 0.01	nd	nd	0.09 ± 0.01
p-Cymene	0.16 ± 0.02	0.24 ± 0.01	0.22 ± 0.03	0.26 ± 0.03	0.25 ± 0.03
Terpinolene	0.27 ± 0.03	0.33 ± 0.01	0.35 ± 0.02	0.53 ± 0.02	0.36 ± 0.02
Terpinen-4-ol	0.11 ± 0.01	0.12 ± 0.01	0.82 ± 0.02	0.10 ± 0.00	0.26 ± 0.06
**Norisoprenoids**					
β-Damascenone	5.46 ± 0.07	5.45 ± 0.92	10.30 ± 0.28	7.93 ± 1.32	9.07 ± 0.06
3-Hydroxy-β-damascone	0.18 ± 0.02	0.16 ± 0.05	0.26 ± 0.01	0.24 ± 0.01	0.14 ± 0.01
Vitispirane	9.77 ± 0.66	10.79 ± 1.84	11.99 ± 3.46	16.45 ± 0.47	13.37 ± 0.69
TPB	0.07 ± 0.01	0.08 ± 0.01	0.18 ± 0.01	0.17 ± 0.01	0.12 ± 0.02
TDN	1.81 ± 0.01	1.76 ± 0.37	3.69 ± 0.21	3.70 ± 0.01	2.78 ± 0.42
**Benzenoids and others**					
Benzyl alcohol	45.93 ± 4.19	35.16 ± 1.95	44.49 ± 2.60	56.89 ± 5.24	72.90 ± 2.55
Vanillin	6.06 ± 0.25	5.94 ± 0.22	5.94 ± 0.06	5.88 ± 0.17	6.04 ± 0.08
Ethyl vanillate	182.83 ± 18.82	172.96 ± 1.77	189.04 ± 7.86	170.44 ± 4.26	191.00 ± 20.58
Methyl vanillate	11.69 ± 0.74	9.33 ± 0.24	14.28 ± 1.34	11.61 ± 0.53	12.30 ± 0.12
Benzaldehyde	14.50 ± 0.22	14.30 ± 0.19	15.03 ± 0.21	14.75 ± 0.00	14.60 ± 0.14
Eugenol	1.90 ± 0.01	1.88 ± 0.13	2.01 ± 0.28	1.88 ± 0.11	1.88 ± 0.11
Methyl salicylate	1.52 ± 0.18	1.57 ± 0.25	3.92 ± 0.01	3.05 ± 0.06	2.48 ± 0.12
2,6-Dimethoxyphenol	5.28 ± 0.06	5.07 ± 0.12	4.83 ± 0.37	5.04 ± 0.39	6.11 ± 0.27
Furfural	0.67 ± 0.07	1.04 ± 0.72	0.97 ± 0.10	0.97 ± 0.04	0.63 ± 0.15
γ-Decalactone	2.47 ± 0.17	3.30 ± 0.28	3.09 ± 0.32	2.32 ± 0.11	2.34 ± 0.02
δ-Decalactone	17.63 ± 3.57	23.47 ± 2.17	20.19 ± 1.21	18.71 ± 0.62	14.87 ± 0.81

Nd means not detected. TPB is short for (E)-1-(2,3,6-Trimethylphenyl)-buta-1,3-diene. TDN is short for 1,6,-trimethyl-1,2-dihydronapthalene.

**Table 6:** Concentration (µg/L) of volatile compounds of Area 2 Corvinone wines.

	Yeast 1	Yeast 2	Yeast 3	Yeast 4	Spontaneous
	mean ± sd	mean ± sd	mean ± sd	mean ± sd	mean ± sd
**Alcohols**					
1-Butanol	435.89 ± 5.62	246.54 ± 7.13	202.54 ± 3.61	161.62 ± 28.98	203.59 ± 32.91
2-Butanol	3865.04 ± 40.39	4224.78 ± 295.96	4888.37 ± 123.11	4074.09 ± 56.97	4692.72 ± 419.96
1-Pentanol	69.68 ± 10.63	53.39 ± 3.74	51.05 ± 1.63	40.72 ± 1.02	36.36 ± 1.58
Isoamyl alcohol (mg/L)	25.80 ± 8.01	32.46 ± 25010	266.04 ± 4.14	312.92 ± 9.35	218.75 ± 2.01
Phenylethyl alcohol (mg/L)	21.67 ± 0.30	26.48 ± 2.34	20.01 ± 0.02	24.69 ± 1.02	15.01 ± 0.06
Methionol	116.97 ± 4.76	217.18 ± 60.02	129.93 ± 9.23	259.07 ± 18.89	69.61 ± 2.59
**C_6_ alcohols**					
1-Hexanol	1149.65 ± 29.59	1424.44 ± 70.95	1054.97 ± 33.81	1154.77 ± 40.13	1189.48 ± 53.31
*trans*-3-Hexen-1-ol	15.01 ± 1.16	19.05 ± 1.02	13.65 ± 0.96	18.31 ± 0.26	13.40 ± 1.28
*cis*-3-Hexen-1-ol	11.12 ± 0.28	15.55 ± 0.16	11.72 ± 0.59	15.26 ± 0.30	13.78 ± 1.94
*cis*-2-Hexen-1-ol	16.00 ± 0.44	15.68 ± 0.33	16.11 ± 0.35	17.28 ± 0.94	15.07 ± 0.94
**Acetate esters**					
Isoamyl acetate	702.55 ± 31.55	689.58 ± 45.25	450.43 ± 15.25	373.92 ± 23.64	708.08 ± 37.99
n-Hexyl acetate	10.75 ± 1.54	12.18 ± 3.84	8.81 ± 2.03	17.77 ± 0.24	14.02 ± 2.14
2-Phenylethyl acetate	26.14 ± 2.51	26.53 ± 2.86	14.21 ± 1.85	14.32 ± 2.01	32.92 ± 2.21
Ethyl acetate (mg/L)	57.70 ± 1.62	70.32 ± 9.73	69.85 ± 1.60	54.02 ± 6.67	158.50 ± 2.59
**Branched-chain fatty acids ethyl esters**					
Ethyl 2-methylbutanoate	1.83 ± 0.04	2.53 ± 0.04	2.31 ± 0.14	2.93 ± 0.09	1.12 ± 0.08
Ethyl 3-methylbutanoate	3.11 ± 0.14	3.10 ± 0.13	3.21 ± 0.16	2.95 ± 0.08	3.10 ± 0.18
**Fatty acids ethyl esters**					
Ethyl butanoate	267.28 ± 27.74	366.95 ± 11.50	201.25 ± 3.67	241.66 ± 12.76	262.34 ± 21.33
Ethyl hexanoate	415.84 ± 14.28	567.81 ± 45.25	358.27 ± 11.13	439.93 ± 23.36	364.84 ± 50.11
Ethyl octanoate	221.11 ± 6.07	320.36 ± 68.82	236.13 ± 6.18	282.17 ± 7.74	198.47 ± 11.00
Ethyl lactate	226.73 ± 7.34	443.77 ± 29.85	206.46 ± 8.03	277.63 ± 36.13	531.84 ± 41.32
Ethyl decanoate	51.72 ± 7.74	85.86 ± 5.85	67.95 ± 2.79	75.81 ± 10.45	48.74 ± 6.30
**Other esters**					
Ethyl 3-hydroxybutanoate	198.08 ± 5.95	266.81 ± 20.89	215.93 ± 5.90	168.82 ± 12.93	128.57 ± 28.39
Ethyl 2-hydroxyhexanoate	0.31 ± 0.04	1.42 ± 0.04	0.66 ± 0.06	0.84 ± 0.06	0.33 ± 0.04
**Fatty acids**					
3-Methylbutanoic acid	374.47 ± 10.27	432.56 ± 57.91	364.05 ± 17.82	459.10 ± 2.18	282.97 ± 14.81
Hexanoic acid	2194.09 ± 377.72	3172.54 ± 401.13	2056.21 ± 65.63	2299.39 ± 149.00	1868.12 ± 27.08
Octanoic acid	4021.06 ± 130.90	5095.48 ± 359.10	3948.40 ± 101.82	4166.92 ± 87.94	3786.14 ± 46.03
**Terpenoids**					
*cis*-Linaloloxide	0.07 ± 0.02	0.11 ± 0.06	0.10 ± 0.01	0.04 ± 0.01	0.08 ± 0.01
*trans*-Linaloloxide	0.08 ± 0.07	0.04 ± 0.00	0.03 ± 0.00	0.08 ± 0.00	0.05 ± 0.01
Linalool	5.34 ± 0.19	6.44 ± 0.05	6.30 ± 0.29	6.52 ± 0.52	5.55 ± 0.66
Geraniol	1.09 ± 0.20	1.67 ± 0.24	1.75 ± 0.21	1.75 ± 0.26	1.68 ± 0.30
α-Terpineol	3.08 ± 0.07	4.18 ± 0.11	4.30 ± 0.32	3.51 ± 0.13	3.41 ± 0.30
β-Citronellol	10.93 ± 0.36	6.57 ± 0.17	7.82 ± 0.84	7.94 ± 0.42	6.92 ± 0.28
α-Phellandrene	2.90 ± 0.16	2.79 ± 0.04	3.07 ± 0.18	3.45 ± 0.35	2.97 ± 0.24
α-Terpinen	0.09 ± 0.01	0.14 ± 0.12	0.05 ± 0.00	0.06 ± 0.01	0.05 ± 0.01
β-Myrcene	3.76 ± 0.21	3.68 ± 0.04	3.98 ± 0.24	4.48 ± 0.45	3.89 ± 0.27
Limonene	0.68 ± 0.05	0.66 ± 0.08	0.71 ± 0.04	0.72 ± 0.04	0.67 ± 0.03
1,4-Cineole	0.04 ± 0.00	nd	nd	nd	nd
1,8-Cineole	0.09 ± 0.00	0.11 ± 0.01	0.10 ± 0.01	0.08 ± 0.01	0.09 ± 0.01
p-Cymene	0.24 ± 0.01	0.16 ± 0.01	0.15 ± 0.00	0.15 ± 0.00	0.17 ± 0.02
Terpinolene	0.33 ± 0.05	0.22 ± 0.03	0.31 ± 0.01	0.29 ± 0.02	0.27 ± 0.03
Terpinen-4-ol	1.59 ± 0.03	0.05 ± 0.01	0.06 ± 0.01	0.05 ± 0.00	0.06 ± 0.01
**Norisoprenoids**					
β-Damascenone	3.19 ± 0.08	2.77 ± 0.15	2.87 ± 0.04	3.03 ± 0.25	2.54 ± 0.23
3-Hydroxy-β-damascone	0.12 ± 0.01	0.15 ± 0.00	0.10 ± 0.01	0.11 ± 0.01	0.15 ± 0.05
Vitispirane	3.56 ± 0.28	4.42 ± 0.14	3.43 ± 0.14	3.54 ± 0.17	3.44 ± 0.23
TPB	0.02 ± 0.00	0.02 ± 0.00	0.02 ± 0.00	0.02 ± 0.00	0.02 ± 0.00
TDN	0.33 ± 0.04	0.34 ± 0.03	0.24 ± 0.01	0.26 ± 0.01	0.26 ± 0.02
**Benzenoids and others**					
Benzyl alcohol	39.17 ± 0.30	34.19 ± 1.61	31.11 ± 3.26	52.14 ± 5.28	56.54 ± 0.81
Vanillin	5.45 ± 0.07	5.54 ± 0.16	5.49 ± 0.21	5.60 ± 0.21	5.50 ± 0.14
Ethyl vanillate	177.78 ± 4.48	185.85 ± 22.39	145.92 ± 19.79	184.34 ± 21.53	180.38 ± 33.66
Methyl vanillate	10.56 ± 0.22	13.24 ± 0.30	11.47 ± 0.72	12.80 ± 1.77	12.04 ± 1.13
Benzaldehyde	14.50 ± 0.09	15.03 ± 0.69	14.19 ± 0.49	14.84 ± 0.24	14.80 ± 0.11
Eugenol	1.26 ± 0.01	1.26 ± 0.01	1.16 ± 0.06	1.26 ± 0.01	1.68 ± 0.01
Methyl salicylate	0.64 ± 0.07	0.77 ± 0.04	0.63 ± 0.13	0.64 ± 0.08	0.37 ± 0.02
2,6-Dimethoxyphenol	6.70 ± 0.42	5.66 ± 0.53	5.11 ± 0.65	6.98 ± 0.03	5.99 ± 0.37
Furfural	1.79 ± 0.08	2.10 ± 0.10	1.62 ± 0.12	1.86 ± 0.19	1.35 ± 0.19
γ-Decalactone	2.25 ± 0.00	3.20 ± 0.16	2.47 ± 0.30	3.02 ± 0.11	2.32 ± 0.08
δ-Decalactone	22.06 ± 0.52	28.68 ± 4.83	16.57 ± 2.34	28.13 ± 2.51	23.11 ± 2.44

Nd means not detected. TPB is short for (E)-1-(2,3,6-Trimethylphenyl)-buta-1,3-diene. TDN is short for 1,6,-trimethyl-1,2-dihydronapthalene.

## Data Availability

Data is contained within the article and supplementary material.

## Supplementary Materials

The following are available online at https://www.mdpi.com/article/10.3390/foods10102474/s1, Table S1: Enological parameters of fresh Corvina and Corvinone musts at crush; Table S2: Quantification methods, retention indices, quantification ions of studied compounds; Figure S1: Fermentation kinetics of each biological replicate (a and b) of all wines; Table S3: Kruskal–Wallis (*p* < 0.05) of volatile compounds according to employed yeasts and grape origin in wines; Table S4: Significantly different compounds according to Kruskal–Wallis analysis (α = 0.05) between Spontaneous and inoculated (yeast 1, yeast 2, yeast 3, yeast 4) fermentations in wines; Table S5: Concentration (µg/L) of significant different volatile compounds among different sensory clusters according to Kruskal–Wallis (α = 0.05).
