# Supplementary material for: Volatile Compounds in Monovarietal Wines of Two Amarone Della Valpolicella Terroirs: Chemical and Sensory Impact of Grape Variety and Origin, Yeast Strain and Spontaneous Fermentation
Source: Foods. 2021 Oct 15;10(10):2474. doi: 10.3390/foods10102474 (PMC8536046; doi:10.3390/foods10102474)
Supplement: Supplementary file 1 [file foods-10-02474-s001.zip › foods-1396524-supplementary.pdf]

Table S1. Enological parameters of fresh Corvina and Corvinone musts at crush

	Glucose+fructose (g/L)			pH		PAN[a] (mg/L)		Ammonia (mg/L)	YAN[b] (mg/L)
	mean	***mean***	***sd***	***mean***	***sd***	***mean***	***sd***	***mean***	***mean***
Area 1 Corvina	199.6 ab	3.13 ab	0.03	92.1 b	7.3	56.1 b	7.5	148.2 b	3.13 ab
Area 2 Corvina	203.1 ab	3.1 a	0.05	84.7 a	3.8	48.8 a	3.7	133.5 a	3.1 a
Area 1 Corvinone	192.5 a	3.17 b	0.03	117.2 d	6.7	77.2 d	6.1	194.4 d	3.17 b
Area 2 Corvinone	207.7 b	3.18 b	0.03	103.6 c	5.9	66.7 c	5.8	170.3 c	3.18 b

[a] PAN: Primary Amino Nitrogen; [b]YAN: Yeast Assimilable Nitrogen. Different letters denote statistically significant difference as obtained by Kruskal Wallis (α=0.05) with Dunn multiple pairwise comparison

Table S2. Extraction methods, retention indices, and quantification ions of studied compounds

	EM[1]	LRI[2]	Identification[3]	Quantitation ion *m/z*	Qualifier ions *m/z*
1-Butanol	a	1159	RS	56,00	55,00
2-Butanol	a	1020	RS	59,00	
1-Pentanol	a	1256	RS	55,00	56, 57, 70
Isoamyl alcohol	a	1220	RS	57,00	55, 56, 70
Phenylethyl alcohols	a	1920	RS	91,00	65, 92, 122
Methionol	a	1719	RS	106,00	57, 61, 73
1-Hexanol	a	1316	RS	56,00	55, 69
trans-3-Hexen-1-ol	a	1379	RS	67,00	55, 69, 82
cis-3-Hexen-1-ol	a	1391	RS	68,00	55, 69, 83
cis-2-Hexen-1-ol	a	1370	RS	57,00	57, 82
Isoamyl acetate	a	1125	RS	70,00	55, 60, 87
n-Hexyl acetate	a	1271	RS	56,00	55, 61, 84
2-Phenethyl acetate	a	1801	RS	104,00	91,00
Ethyl acetate	b	890	LRI MS	61,00	70, 88
Ethyl 2-methyl butanoate	a	1040	RS	102,00	74, 85, 115
Ethyl 3-methyl butanoate	a	1069	RS	88,00	57, 60, 85
Ethyl 3-hydroxybutanoate	a	1506	RS	117,00	71, 87
Ethyl di-2-hydroxyhexanoate	a	1540	LRI MS	87,00	69, 104
Ethyl butanoate	a	1032	RS	71,00	88,00
Ethyl hexanoate	a	1240	RS	88,00	60, 99
Ethyl octanoate	a	1430	RS	88,00	57, 100, 127
Ethyl decanoate	a	1640	RS	88,00	71, 101, 155
3-Methylbutanoic acid	a	1667	RS	60,00	87,00
Hexanoic acid	a	1839	RS	60,00	73, 87
Octanoic acid	a	2071	RS	60,00	73, 101, 115
cis-Linalooloxide	b	1437	RS	59,00	111, 94
trans-Linalooloxide	b	1469	RS	59,00	111, 94
Linalool	b	1547	RS	71,00	121, 93
Geraniol	b	1860	RS	93,00	123, 121, 105
β-Citronellol	b	1771	RS	69,00	82, 81, 67
α-Terpineol	b	1701	RS	136,00	121, 93, 59
α-Phellandrene	b	1180	RS	93,00	136, 91
α-Terpinen	b	1188	RS	121,00	93, 126
β-Myrcene	b	1161	RS	93,00	69, 79
Limonene	b	1198	RS	136,00	139, 125, 111
1,4-Cineole	b	1186	RS	154,00	139, 125, 111
1,8-Cineole	b	1217	RS	154,00	139, 111, 108
p-Cymene	b	1271	RS	119,00	134, 91

Terpinolene	b	1283	RS	121,00	136, 93
Terpinen-4-ol	b	1614	RS	71,00	111, 93, 86
β-Damascenone	b	1825	RS	69,00	190, 121, 105
3-Oxo-α-ionol	a	2555	LRI MS	108,00	152,00
3-Hydroxy-β-damascone	a	2535	LRI MS	69,00	175, 193, 208
Vitispirane	b	1523	LRI MS	192,00	177, 93
TPB	b	1828	LRI MS	172,00	157, 142
TDN	b	1745	LRI MS	157,00	172, 142
Benzyl Alcohol	a	1874	RS	106,00	105, 77, 51
Vanillin	a	2572	RS	151,00	81, 152, 109
Ethyl vanillate	a	2665	RS	151,00	168, 196
Methyl vanillate	a	2630	RS	151,00	123, 182
Benzaldehyde	a	1538	RS	106,00	51, 77, 105
Eugenol	b	2172	RS	164,00	103, 149
Methyl salicylate	a	1771	RS	121,00	92, 152
γ-Decalactone	a	2141	RS	85,00	128,00
δ-decalactone	a	2193	RS	99,00	55, 71
2,6-Dimethoxyphenol	a	2270	RS	154,00	95, 111, 139
Furfural	a	1474	RS	96,00	95,00

1 Extraction method: a (SPE) and b (SPME)

2 Linear Retention Index (LRI) were determined on DB-WAX polar column.

3 RS identified using reference standard; LRI MS tentatively identified by comparing the Linear Retention Index and mass spectra with those of literature.

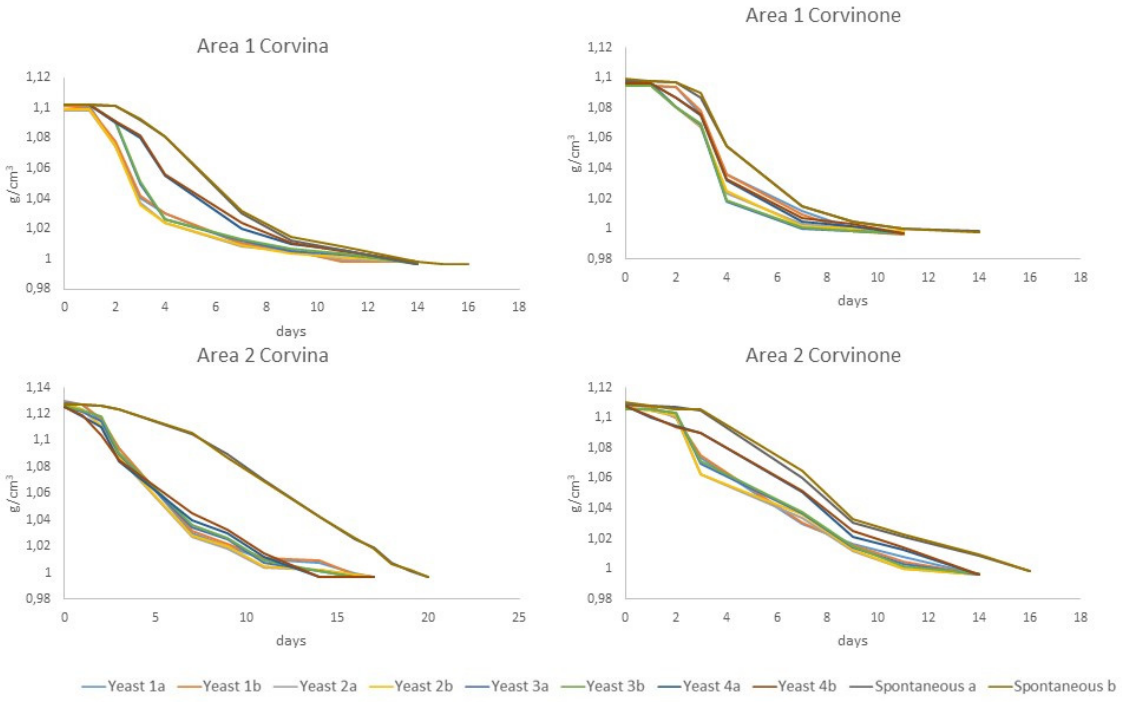

Figure S1. Fermentation kinetics of each biological replicates (a and b) of all wines

Table S3. Kruskal Wallis ($p<0,05$) of volatile compounds according to employed yeasts and grape origin in wines.

	Corvina		Corvinone	
	**Yeast**	**Area**	**Yeast**	**Area**
	S[a]	*S*[a]	*S*[a]	*S*[a]
**Alcohols**				
1-Butanol	**Yes**	No	**Yes**	No
2-Butanol	**Yes**	No	No	**Yes**
1-Pentanol	No	No	No	No
Isoamyl alcohol	No	No	**Yes**	No
Phenylethyl Alcohol	No	No	**Yes**	No
Methionol	No	No	**Yes**	No
**C_6 alcohols**				
1-Hexanol	No	**Yes**	No	**Yes**
trans-3-Hexen-1-ol	No	**Yes**	No	**Yes**
cis-3-Hexen-1-ol	No	**Yes**	No	**Yes**
cis-2-Hexen-1-ol	No	No	No	No
**Acetate esters**				
Isoamyl acetate	No	**Yes**	**Yes**	No
n-Hexyl acetate	**Yes**	No	No	No
2-Phenethyl acetate	No	No	**Yes**	**Yes**
Ethyl acetate	**Yes**	No	**Yes**	No
**Branched-chain fatty acids ethyl esters**				
Ethyl-2-methylbutanoate	**Yes**	No	**Yes**	No
Ethyl 3-methylbutanoate	No	**Yes**	No	No
**Fatty acids ethyl esters**				
Ethyl butanoate	**Yes**	No	No	**Yes**
Ethyl hexanoate	**Yes**	No	**Yes**	**Yes**
Ethyl octanoate	**Yes**	No	No	**Yes**
Ethyl decanoate	**Yes**	No	No	**Yes**
Ethyl lactate	**Yes**	No	**Yes**	**Yes**
**Other esters**				
Ethyl 3-hydroxybutanoate	No	**Yes**	**Yes**	No
Ethyl 2-hydroxyhexanoate	No	**Yes**	**Yes**	No
**Fatty acids**				
3-Methylbutanoic acid	No	**Yes**	No	No
Hexanoic acid	**Yes**	No	**Yes**	No
Octanoic acid	**Yes**	No	**Yes**	No
**Terpenoids**				
cis-Linaloloxide	**Yes**	No	No	No
trans-Linaloloxide	**Yes**	No	No	No
Linalool	No	**Yes**	No	**Yes**
Geraniol	**Yes**	No	No	**Yes**
α-Terpineol	No	**Yes**	**Yes**	No
β-citronellol	No	**Yes**	No	**Yes**
α-Phellandrene	No	**Yes**	No	**Yes**
α-Terpinen	No	**Yes**	No	**Yes**
β-Myrcene	No	**Yes**	No	**Yes**
Limonene	No	**Yes**	No	No
1,4-Cineol	**Yes**	No	**Yes**	No

1,8-Cineol	No	No	No	**Yes**
p-Cymene	No	No	No	**Yes**
Terpinolene	No	**Yes**	No	**Yes**
Terpinen-4-ol	**Yes**	No	No	**Yes**
**Norisoprenoids**				
β-damascenone	No	**Yes**	No	**Yes**
3-Hydroxy-β-damascone	No	**Yes**	No	**Yes**
Vitispirane	No	**Yes**	No	**Yes**
TPB	**Yes**	No	No	**Yes**
TDN	No	**Yes**	No	**Yes**
**Benzenoids and others**				
Benzyl alcohol	No	**Yes**	**Yes**	No
Vanillin	No	**Yes**	No	**Yes**
Ethyl-vanillate	No	**Yes**	No	No
Methyl-vanillate	**Yes**	No	No	No
Benzaldehyde	No	**Yes**	No	No
Eugenol	No	**Yes**	No	**Yes**
Methyl salycilate	No	**Yes**	No	**Yes**
2,6-Dimethoxy-phenol	No	No	No	**Yes**
Furfural	No	**Yes**	No	**Yes**
γ-decalactone	No	No	**Yes**	No
δ-decalactone	No	**Yes**	No	**Yes**

Table S4. Significantly different compounds according to Kruskal Wallis analysis (α=0.05) between Spontaneous and inoculated (Yeast 1, yeast 2, yeast 3, yeast 4) fermentations in wines.

	Area 1 Corvina	Area 2 Corvina	Area 1 Corvinone	Area 2 Corvinone
**Alcohols**	*S*[a]	*S*[a]	*S*[a]	*S*[a]
1-Butanol	**Yes**	No	No	No
2-Butanol	No	**Yes**	**Yes**	No
1-Pentanol	**Yes**	No	0,192	**Yes**
Isoamyl alcohol	**Yes**	No	**Yes**	**Yes**
Phenylethyl Alcohol	**Yes**	No	**Yes**	**Yes**
Methionol	No	No	**Yes**	**Yes**
**C_6 alcohols**				
1-Hexanol	**Yes**	No	No	No
trans-3-Hexen-1-ol	**Yes**	No	**Yes**	No
Acetate esters				
Isoamyl acetate	**Yes**	No	No	No
n-Hexyl acetate	No	No	No	No
2-Phenethyl acetate	**Yes**	No	**Yes**	**Yes**
Ethyl acetate	**Yes**	**Yes**	**Yes**	**Yes**
**Branched-chain fatty acids ethyl esters**				
Ethyl-2-methylbutanoate	**Yes**	**Yes**	**Yes**	**Yes**
Ethyl 3-methylbutanoate	**Yes**	No	No	No
**Fatty acids ethyl esters**				
Ethyl butanoate	**Yes**	No	**Yes**	No
Ethyl hexanoate	**Yes**	No	**Yes**	No
Ethyl octanoate	**Yes**	No	**Yes**	**Yes**
Ethyl decanoate	No	No	**Yes**	No
Ethyl lactate	**Yes**	**Yes**	**Yes**	**Yes**
**Other esters**				
Ethyl 3-hydroxybutanoate	**Yes**	No	**Yes**	**Yes**
Ethyl 2-hydroxyhexanoate	No	No	**Yes**	No
**Fatty acids**				
3-Methylbutanoic acid	**Yes**	No	No	**Yes**
Hexanoic acid	**Yes**	No	**Yes**	**Yes**
Octanoic acid	**Yes**	No	**Yes**	**Yes**
**Terpenoids**				
cis-Linaloloxide	**Yes**	No	No	No
trans-Linaloloxide	No	No	**Yes**	No
α-Terpineol	No	**Yes**	No	No
α-Phellandrene	No	No	**Yes**	No
Limonene	No	**Yes**	No	No
1,8-Cineol	No	**Yes**	No	No
Terpinolene	No	**Yes**	No	No
Norisoprenoids				
β-damascenone	No	**Yes**	No	No
Vitispirane	No	**Yes**	No	No
TPB	**Yes**	**Yes**	No	No
TDN	No	**Yes**	No	No
**Benzenoids and others**				
Benzyl alcohol	**Yes**	**Yes**	**Yes**	**Yes**
Vanillin	**Yes**	No	No	No
Methyl-vanillate	No	**Yes**	No	No
Benzaldehyde	**Yes**	No	No	No
Eugenol	**Yes**	No	No	**Yes**
Methyl salycilate	**Yes**	No	No	**Yes**
2,6-Dimethoxy-phenol	No	No	**Yes**	No
Furfural	**Yes**	No	No	**Yes**
δ-decalactone	**Yes**	No	No	No

Table S5. Concentration (µg/L) of significant different volatile compounds among different sensory clusters according to Kruskal Wallis (α=0.05)

	p-value	Cluster 1 mean (µg/L)	Cluster 2 mean (µg/L)	Cluster 3 mean (µg/L)
**Corvina**				
2-Butanol	0,032	5034,92 ab	5022,67 b	4035,39 a
Isoamyl alcohol (mg/L)	0,004	27,06 b	17,7 a	22,12 a
1-Hexanol	0,001	914,69 b	765,53 a	782,65 a
trans-3-Hexen-1-ol	0,001	10,2 b	6,8 a	7,26 a
cis-3-Hexen-1-ol	0,014	42,18 b	39,88 ab	37,69 a
Isoamyl acetate	0,002	353,05 ab	559,02 b	279,76 a
Ethyl acetate (mg/L)	0,006	62,12 a	140,86 b	53,85 a
Ethyl 2-methylbutanoate	0,007	3,17 b	1,35 a	3,39 b
Ethyl 3-methylbutanoate	0,008	2,75 a	2,51 ab	3,98 b
Ethyl butanoate	0,010	279,5 b	133,9 a	202,08 ab
Ethyl hexanoate	0,017	400,63 b	221,2 a	311,8 ab
Ethyl lactate	0,010	237,32 a	348,09 b	230,25 a
Ethyl 3-hydroxybutanoate	0,001	190,87 b	81,06 a	95,66 a
Ethyl 2-hydroxyhexanoate	0,043	1,04 b	0,45 ab	0,42 a
Ethyl decanoate	0,041	41,88 ab	27,22 a	59,79 b
Acetic acid	0,008	0,34 a	0,89 b	0,29 a
Hexanoic acid	0,016	2129,3 b	1139,98 a	1660,45 ab
3-carene	0,041	0,06 a	0,07 ab	0,08 b
α-Phellandrene	0,006	2,86 a	4,95 ab	7,15 b
β-Myrcene	0,003	4,18 a	6,75 ab	9,26 b
Limonene	0,001	0,74 a	1,02 ab	1,68 b
1,8-Cineole	0,039	0,07 a	0,08 ab	0,12 b
Terpinolene	0,001	0,51 a	0,63 a	1,04 b
Linalool	0,010	5,9 a	6,94 ab	9,83 b
α-Terpineol	0,001	3,36 a	3,39 a	6,55 b
β-Damascenone	0,004	7,17 b	4,12 a	3,51 a
TPB	0,017	0,04 b	0,02 a	0,03 ab
TDN	0,027	0,67 b	0,35 a	0,38 a
3-Hydroxy-β-damascone	0,018	0,31 b	0,3 ab	0,19 a
Benzyl alcohol	0,000	300,16 b	254,05 ab	212,47 a
Eugenol	0,001	7,18 b	6,22 a	5,86 a
Vanillin	0,001	5,5 b	3,44 a	4,92 a
Ethyl vanillate	0,003	123,67 a	135,13 ab	139,27 b
Methyl salycilate	0,002	0,87 a	1,37 a	3,67 b
δ-Decalactone	0,004	30,8 b	23,64 a	24 a
**Corvinone**				
1-Hexanol	0,002	1849,98 b	1152,21 a	1344,73 a
trans-3-Hexen-1-ol	0,001	27,82 b	16,66 a	15,84 a
cis-3-Hexen-1-ol	0,005	19,17 b	13,19 a	15,04 a
Ethyl acetate (mg/L)	0,023	68,2 a	55,86 a	114,62 b
Acetic acid	0,010	0,53 b	0,22 a	0,61 b
α-Phellandrene	0,001	1,15 a	3,17 b	2,75 b
β-Myrcene	0,001	2,02 a	4,12 b	3,6 b
1,8-Cineole	0,018	0,04 a	0,09 ab	0,09 b
Linalool	0,003	3,86 a	5,93 b	5,83 b
β-Citronellol	0,014	3,82 a	5,28 b	9,43 a
β-Damascenone	0,021	6,21 b	7,28 a	3,11 a
TPB	0,005	0,12 b	0,02 a	0,04 a
TDN	0,008	2,74 b	0,3 a	0,9 a
3-Hydroxy-β-damascone	0,004	0,21 b	0,11 a	0,13 a
Vitispirane	0,010	12,25 b	3,55 a	6,16 a
Furfural	0,018	0,91 a	1,82 b	1,42 ab
Eugenol	0,005	1,91 b	1,26 a	1,49 a
2,6-Dimethoxyphenol	0,002	5,05 a	6,84 c	5,72 b
Vanillin	0,014	5,95 b	5,52 a	5,64 a
Methyl salycilate	0,007	2,51 b	0,64 a	1,06 a

Different letters in the same row denote statistically significant difference as obtained by Kruskal Wallis (α=0.05) with Dunn multiple pairwise comparison
